# Avocado Seeds Relieve Oxidative Stress-Dependent Nephrotoxicity but Enhance Immunosuppression Induced by Cyclosporine in Rats

**DOI:** 10.3390/antiox10081194

**Published:** 2021-07-27

**Authors:** Amira M. Elmoslemany, Mohammed A. El-Magd, Heba I. Ghamry, Mohammad Y. Alshahrani, Nahla S. Zidan, Amina M. G. Zedan

**Affiliations:** 1 Nutrition and Food Science Department, Faculty of Home Economic, Al Azhar University, Tanta 31732, Egypt; amiraelmoslemany@azhar.edu.eg; 2 Department of Anatomy, Faculty of Veterinary Medicine, Kafrelsheikh University, Kafrelsheikh 33516, Egypt; 3 Department of Home Economics, College of Home Economics, King Khalid University, Abha 61413, Saudi Arabia; hgmry@kku.edu.sa; 4 Research Center for Advanced Materials Science (RCAMS), King Khalid University, Abha 61413, Saudi Arabia; moyahya@kku.edu.sa; 5 Department of Clinical Laboratory Sciences, College of Applied Medical Sciences, King Khalid University, Abha 9088, Saudi Arabia; 6 Department of Nutrition and Food Science, Faculty of Home Economics, Tabuk University, Tabuk 47512, Saudi Arabia; nzidan@ut.edu.sa; 7 Department of Home Economics, Faculty of Specific Education, Kafrelsheikh University, Kafrelsheikh 33516, Egypt; 8 Biological and Environmental Sciences Department, Faculty of Home Economic, Al Azhar University, Tanta 31732, Egypt; AminaZedan1948.el@Azhar.edu.eg

**Keywords:** avocado seeds, cyclosporine A, nephrotoxicity, immunosuppression, oxidative stress

## Abstract

Cyclosporine A’s (CsA) immunosuppressive effect makes it an ideal drug for organ transplantation. However, CsA’s uses are restricted due to its side effects. We investigated the effects of avocado seed (AvS) powder on CsA-induced nephrotoxicity and immunosuppression in rats. The injection of CsA (5 mg/kg, subcutaneously, for 10 days) increased serum levels of creatinine, uric acid, and urea, and the renal levels of the malondialdehyde. It decreased creatinine clearance and the renal activity of antioxidant enzymes (superoxide dismutase, catalase, and glutathione peroxidase) and Na^+^/K^+^ ATPase. The administration of CsA also significantly downregulated the renal expression of interferon-gamma, tumor necrosis factor-alpha, interleukin 1 beta, monocyte chemotactic protein 1, intercellular adhesion molecule-1, and vascular cell adhesion molecule 1 genes, and increased renal DNA damage. Histopathological examination confirmed the biochemical and molecular alterations that accompanied CsA nephrotoxicity. All CsA-induced deleterious effects, except immunosuppression, were ameliorated by feeding rats on a basal diet supplemented with 5% AvS powder for 4 weeks. Importantly, AvS also maximized CsA’s immunosuppressive effect. These findings suggest a potential ameliorative effect of AvS on CsA-induced nephrotoxicity, and AvS enhances CsA’s immunosuppressive effect. Therefore, AvS might be used in combination with CsA in transplantation treatment to relieve the CsA-induced nephrotoxicity.

## 1. Introduction

Cyclosporine (CsA) is an antibiotic extracted from *Tolypocladium inflatum gams* fungus that possesses powerful immunosuppressive properties which make it an ideal drug for solid organ transplantations (particularly for the kidneys) [1]. Administration of CsA to patients after solid organ transplantation improves survival statistically [2,3]. However, CsA’s prolonged use results in oxidative-stress-dependent side effects in many organs, including severe nephrotoxicity [4,5,6]. Indeed, some patients who underwent heart transplantations and were post-surgically treated with CsA for a long time suffered from nephrotoxicity [7]. Specifically, CsA can induce oxidative-stress-dependent nephrotoxicity by triggering endoplasmic reticulum stress and boosting free radical release from mitochondria [8]. Another mechanism by which CsA could cause nephrotoxicity is the induction of apoptosis in renal cells [9]. Due to its importance after transplantation surgeries, there is an urgent need for researchers to find out safe adjuvants to reduce CsA’s side effects without inhibiting its immunosuppressive potential. 

The avocado (*Persea americana* Mill.), a member of the Lauraceae flowering plant family, has very high levels of unsaturated oils and high nutritional value [10]. Avocado pulp is rich in many health promoting constituents, such as carotenoids (especially lutein); monounsaturated fatty acids (particularly oleic acid); and phenolic compounds, such as hydroxybenzoic acid, hydrocinnamic acid, and catechin [11,12]. These bioactive constituents have antioxidant, anti-inflammatory, anticancer, antimicrobial, antidiabetic, and hypolipidemic properties [13]. In decades past, the avocado seed (AvS), which represents 13–18% of the fruit, was considered a waste product and was discarded during the processing of the pulp, causing serious ecological concern [14]. In the last decade, researchers investigated the chemical structure of the AvS and found several health-promoting bioactive compounds, including phytosterols, triterpenes, fatty acids, furanoic acids, proanthocyanidins, and abscisic acid [15]. Moreover, the AvS has higher amounts of phenolic compounds (such as hydrocinnamic acid, catechin, epicatechin, and leucoanthocyanidins) than the pulp [16]. These health-promoting bioactive compounds give the AvS many medicinal properties, including antioxidant, anticancer, antimicrobial, antifungal, anthelmintic, hypotensive, antidiabetic, and hypolipidemic properties [17,18,19]. Among the extracts of 15 fruit wastes investigated for their antioxidant properties, the AvS showed the most potent activity [20]. For their health-promoting and nutritious properties, AvS extracts could serve as an ingredient in functional foods. A recent study by Saad [21] showed that feeding rats on a basal diet supplemented with 5% AvS powder can improve immunity following treatment with CsA. In this study, the author used a very high dose of CsA (50 mg/kg, subcutaneously, for 10 days) instead of the optimal dose (5 mg/kg). Moreover, Saad’s study lacked a molecular approach to confirm their hematological and biochemical findings.

Based on the aforementioned properties of the AvS, it seems to have much ecological and economic potential, and might be able to be used in the treatment of oxidative stress and inflammatory-related diseases and toxicities. However, the AvS’s effects on CsA-induced nephrotoxicity and immunosuppression remained elusive prior to our study’s commencement. Therefore, this study aimed to explore the ameliorative potential of the AvS for CsA-induced nephrotoxicity by investigating the modulations of oxidative stress, apoptosis, and inflammation associated with CsA-induced nephrotoxicity. 

## 2. Materials and Methods

### 2.1. Plant and Chemicals

Avocados used in this study were obtained from a local market. Cyclosporine-A (Sandimun Neoral^®^) was purchased in form of capsules (25 mg/capsule) from Novartis Pharma Co., Plantation, FL, USA.

### 2.2. Diet Preparation

The basal diet of the rats was prepared following the laboratory animal diet guidelines as previously described [22]. The avocado seeds (AvS) were dried in an oven (Contherm Thermotec 2000, Taipei, Taiwan) at 40 °C for 48 h until achieving a moisture level of 4%. The small pieces were then crushed into powder with a Retsch laboratory mill and passed through a 40 mesh screen to obtain avocado seed powder. Then the powder was stored in plastic packs at 5 °C for further analyses. Treatment diets were prepared by incorporating 50 g of AvS powder into each kilogram of food to get a concentration of 5%. The formulations of the basal and treatment diets are shown in Table 1. 

### 2.3. Detection of Bioactive Compounds by GC-MS

Bioactive compounds in AvS powder were detected by gas chromatography coupled to mass spectrometry (GC-MS) by using an ISQ LT single quadrupole mass spectrometer (Thermo Scientific TRACE 1310 Gas Chromatograph, Walham, MA, USA). The ethanolic extract of the AvS was used for the GC-MS analysis.

### 2.4. Experimental Design

Twenty-eight Sprague–Dawley male rats weighing 120–160 g which were 10 to 12 weeks old were purchased from the Vaccine and Immunity Organization, Ministry of Health, Egypt. The animals were housed in cages supplied with a light/dark system of 12 h/12 h at 23 ± 2 °C. They were fed a basal diet ad libitum and had free access to water. The animals were acclimatized for one week; then they were randomized into four groups. Each group contained seven animals. In the first group (control group; Cnt), rats were given a basal diet for 4 weeks and were subcutaneously injected with 200 µL/rat/daily of olive oil as a vehicle for 10 days (from D1 to D10). In the second group (avocado seeds—AvS), rats were fed on a basal diet supplemented with 5% AvS powder for 4 weeks. In the third group (cyclosporine A—CsA), rats were subcutaneously injected with 5 mg/kg/day CsA in olive oil for 10 days and were fed on a basal diet for 4 weeks. Each CsA capsule (25 mg) was dissolved in 5 mL of olive oil, and each animal was injected with 200 µL of this mixture as previously described [22]. In the fourth group (CsA + AvS), rats received CsA at a dose of 5 mg/kg/day subcutaneously for 10 days and were fed on a basal diet supplemented with 5% AvS powder for 4 weeks. Ethical approval from the Animal Ethical Committee of Kafrelsheikh University, license number KFS-127/29, was obtained to perform this experiment.

### 2.5. Growth-Related Parameters

Feed intake (FI) was recorded weekly. Growth-related parameters, including the final body weight gain (FBWG) and feed efficiency ratio (FER), were determined using the following equations: FBWG (g) = final BW (g) − initial BW (g); FER = FBWG (g)/total FI (g).

### 2.6. Sampling

At the end of the experiment, rats were placed in metabolic cages individually to collect the 24 h urine totals to determine creatinine clearance. The animals were fasted overnight before being euthanized by exsanguination. Blood samples were collected from jugular veins and sera were separated by centrifugation at 4000 rpm for 10 min. The kidneys were immediately removed, washed with isotonic saline, and weighed. The kidney was split into 4 parts: the first part was frozen at −80 °C for isolation of total RNA and further molecular analysis, the second part was fixed in 10% formalin for histopathological examination, the third part was freshly used for the comet assay, and the fourth part was homogenized for antioxidant analysis.

### 2.7. Biochemical Analysis

Serum levels of uric acid, urea, and creatinine were determined according to methods described by Fossati et al. [23], Patton and Crouch [24], and Bartels et al. [25], respectively. Creatinine clearance was estimated from the levels of both serum and urine creatinine in addition to the 24 h urinary volumes. Kidney specimens from all groups were homogenized (1 g tissue + 9 mL homogenization buffer) and centrifuged (1000 rpm for 20 min), and the obtained sediment was used for estimation of Na + K + ATPase using the method described by Bontin [26]. However, the supernatant was used for estimating the levels of the lipid peroxidation marker malondialdehyde (MDA) and the activities of the antioxidant enzymes (superoxide dismutase (SOD; EC 1.15.1.1), catalase (CAT; EC 1.11.1.6), and glutathione peroxidase (GPx; EC 1.11.1.9)). MDA renal levels were assessed as previously described [27]. This method depends on the measurement of thiobarbituric acid reactive substance content (TBARS), which reacts with MDA to produce thiobarbituric acid reactive products, which were measured at the absorbance of 530 nm. SOD activity was assessed as previously described [28]. The method involved blocking epinephrine’s oxidation into adrenochrome, which is hampered by SOD. CAT activity was determined spectrophotometrically at 240 nm by calculating the degradation rate of H_2_O_2_ [29]. GPx activity was determined as previously described [30].

### 2.8. Comet Assay

Comet assays were performed to determine DNA damage in kidneys following different treatments. This assay was carried out as previously described [31]. To obtain separate whole renal cells, fragments of renal tissue (5 mm cubes) were minced using a pair of scissors in chilled PBS supplemented with 54 mM EDTA, and the mixture was filtered using a nylon mesh with a diameter of 150 µm. Based on this assay, DNA damage was quantitatively and qualitatively assessed in a total of 100 cells/sample with the aid of a fluorescence microscope (at 40×), Komet 5 image analysis software (Kinetic Imaging, Ltd., Liverpoo1, UK), and GelRed stain. The DNA damage parameters included the percentage of damaged DNA that migrated away from the intact DNA and tail moment, in addition to the comet tail length, which was measured from the middle of the intact DNA to the end of the tail.

### 2.9. Molecular Analysis by Real-Time PCR

Real-time PCR (qRT-PCR) was used to detect the renal expression levels of immunomodulatory and proinflammation-related genes (interferon-gamma (*IFN-γ*), tumor necrosis factor-alpha (*TNFa*), interleukin 1 beta (*IL1β*), monocyte chemotactic protein 1 (*MCP1*), intercellular adhesion molecule-1 (*ICAM1*), and vascular cell adhesion molecule 1 *(VCAM1*)). To perform qRT-PCR, we first extracted total RNA from kidney tissue using GeneJET RNA Purification Kit following the manufacturer’s protocol (Thermo Scientific, #K0731, Walham, MA, USA). Following the assessment of RNA integrity and purity, a reverse transcriptase (RevertAid H Minus, Thermo Scientific, #EP0451, Walham, MA, USA) was used to convert RNA into cDNA that was used as a template for qRT-PCR reaction in the presence of Syber green master mix (2× Maxima, Thermo Scientific, #K0221, Walham, MA, USA) and gene-specific primers (Table 2). The relative expression was expressed as fold change against the housekeeping *GAPDH* gene using the 2^−ΔΔCt^ method. 

### 2.10. Histopathology Examinations

Following fixation in 10% formalin, kidney specimens were histologically prepared according to standard histological procedures. Sections 4–6 μm thick were stained with hematoxylin and eosin. The kidney damage score in the renal corpuscle involved checking for widened Bowman’s capsules, and shrunken and branched glomeruli; tubular dilatation, pyknotic nuclei, interstitial, and perivascular edema in renal tubules; and hyaline cast in the lumen. This damage score was blindly determined in 5 randomly chosen fields per slide using X400. The score grades were 0 (no damage), 1 (≤10% damage), 2 (11–25% damage), 3 (26–45% damage), 4 (46–75% damage), and 5 (≥76% damage) [32,33]. 

### 2.11. Statistical Analysis

The obtained results were statistically analyzed using one-way ANOVA with GraphPad Prism-8 followed by Tukey’s honestly significant difference. Data are presented as mean ± standard error of mean (SEM) and significance was declared at *p* < 0.05.

## 3. Results

### 3.1. GC-MS Analysis of Avocado Seed Extract

**Figure 1:**
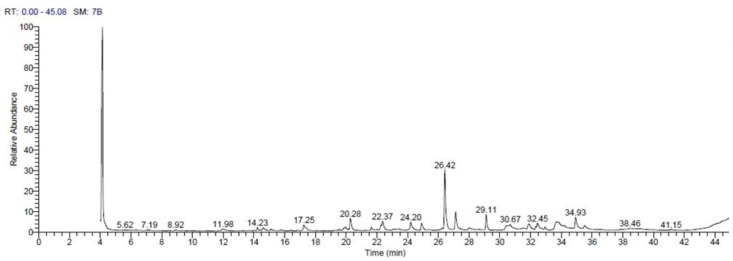
The GC-MS chromatogram of the ethanolic avocado seed extract shows the structures of various phytochemicals at retention times (RT) 4–44 min.

The GC-MS revealed the presence of 21 compounds in the ethanolic extract of the avocado seeds (Table 3, Figure 1). Most of these compounds were terpenes and esters of fatty acid derivatives (dodecanoic acid, tetradecanoic acid, 9,12-octadecadienoic acid (z, z), 9-octadecenoic acid (z), heptadecanoic acid, 16-methyl,-methyl ester, 9,12-octadecadienoic acid, hexadecadienoic acid, and oleic acid), which are phytochemical agents in various plants. 

### 3.2. Growth-Related Parameters and Biochemical Analysis

The CsA group showed significantly (*p* < 0.05) less final body weight gain (FBWG), a significantly lower total feed intake (FI), and a significantly lower feed efficiency ratio (FER) than the control group (Table 4). In contrast, the AvS + CsA group exhibited significantly (*p* < 0.05) higher FBWG, FI, and FER than the CsA group. Surprisingly, rats fed a diet supplemented with 5% AvS powder (AvS group) showed significantly (*p* < 0.05) higher FBWG and FER than the other groups.

Importantly, the AvS group showed significantly lower serum levels of kidney function biomarkers (uric acid, urea, and creatinine) relative to the control (Cnt) group (Table 5). In contrast, rats in the CsA group exhibited significantly (*p* < 0.05) higher serum levels of these biomarkers compared to Cnt and AvS groups. After treatment with AvS (AvS + CsA group), their levels were significantly (*p* < 0.05) declined in comparison to the CsA group.

Rats in the AvS group had significantly lower levels of the lipid peroxidation biomarker MDA and significantly higher levels of GPx in their kidneys when compared with the control (Cnt) group (Table 6). On the other hand, in the CsA group, the levels of MDA were significantly increased, and the activities of the antioxidant enzymes (SOD, CAT, and GPx) were significantly decreased as compared to Cnt and AvS groups. After administration of AvS (AvS + CsA group), MDA levels were significantly reduced and the activities of the antioxidant of enzymes were significantly increased compared to the AvS group.

The CsA group showed significant decreases in creatinine clearance rate in urine, renal Na^+^/K^+^ ATPase activity, and kidney weight compared to control and AvS groups (Table 7). In contrast, the AvS cotreated (AvS + CsA) group significantly increased creatinine clearance, renal Na^+^/K^+^ ATPase, and kidney weight relative to the CsA group. The AvS group exhibited significantly lower creatinine clearance and significantly higher Na^+^/K^+^ ATPase than the AvS + CsA group.

### 3.3. Comet Assay Results

**Figure 2:**
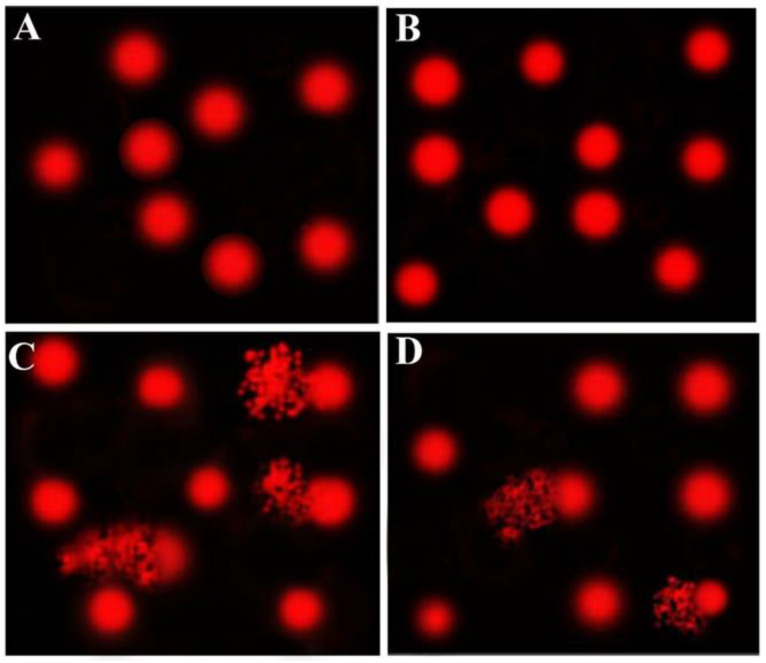
The effects of AvS and/or CsA on the DNA damage of renal cells as detected by comet assay. Comet assays were performed in three independent experiments, and the images here are representative. (**A**) Cnt, (**B**) AvS, (**C**) CsA, (**D**) AvS + CsA. Degraded DNA appears as fragmented patches behind the intact DNA (circle), as seen in (**C**,**D**).

Comet assays were performed to assess the DNA damage in the kidneys after various treatments. The results of the comet assays are shown in Table 8 and Figure 2. The CsA group showed significantly (*p* < 0.05) higher DNA damage in renal cells, as indicated by increases in the tail length, tail DNA%, and tail moment, compared to control and AvS groups. This elevated DNA damage was decreased following cotreatment with AvS (AvS + CsA group). However, no significant difference was noticed between the control and AvS groups.

### 3.4. Molecular Analysis

**Figure 3:**
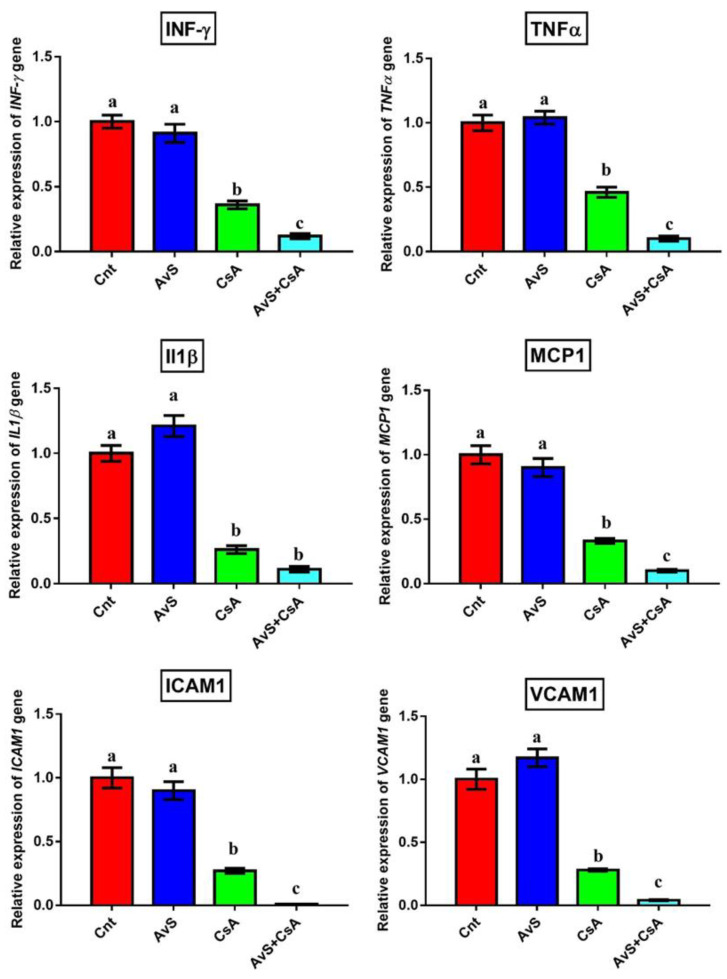
The effects of AvS and/or CsA treatment on the levels of *IFN-γ*, *TNFα*, *IL1β*, *MCP1*, *ICAM1*, and *VCAM1* in rat kidneys, as detected by qRT-PCR. Data are presented as fold change (mean) ± SEM (*n* = 7/group). Columns with different letters (a–c) are significantly different at *p* < 0.05.

To investigate whether AvS could modulate the immunosuppressive effect of CsA, we detected the changes in the expression of immunomodulatory and inflammatory genes (*IFN-γ*, *TNFα*, *IL1β*, *MCP1*, *ICAM1*, and *VCAM1*) in the kidney following treatment with AvS and/or CsA using qRT-PCR. We found significant downregulations in gene expression after treatment with CsA alone or in combination with AvS. The lowest expression levels of such genes were in the co-treated group (AvS + CsA) (Figure 3). On the other hand, no significant difference was found between the control and AvS groups.

### 3.5. Histopathological Examination

**Figure 4:**
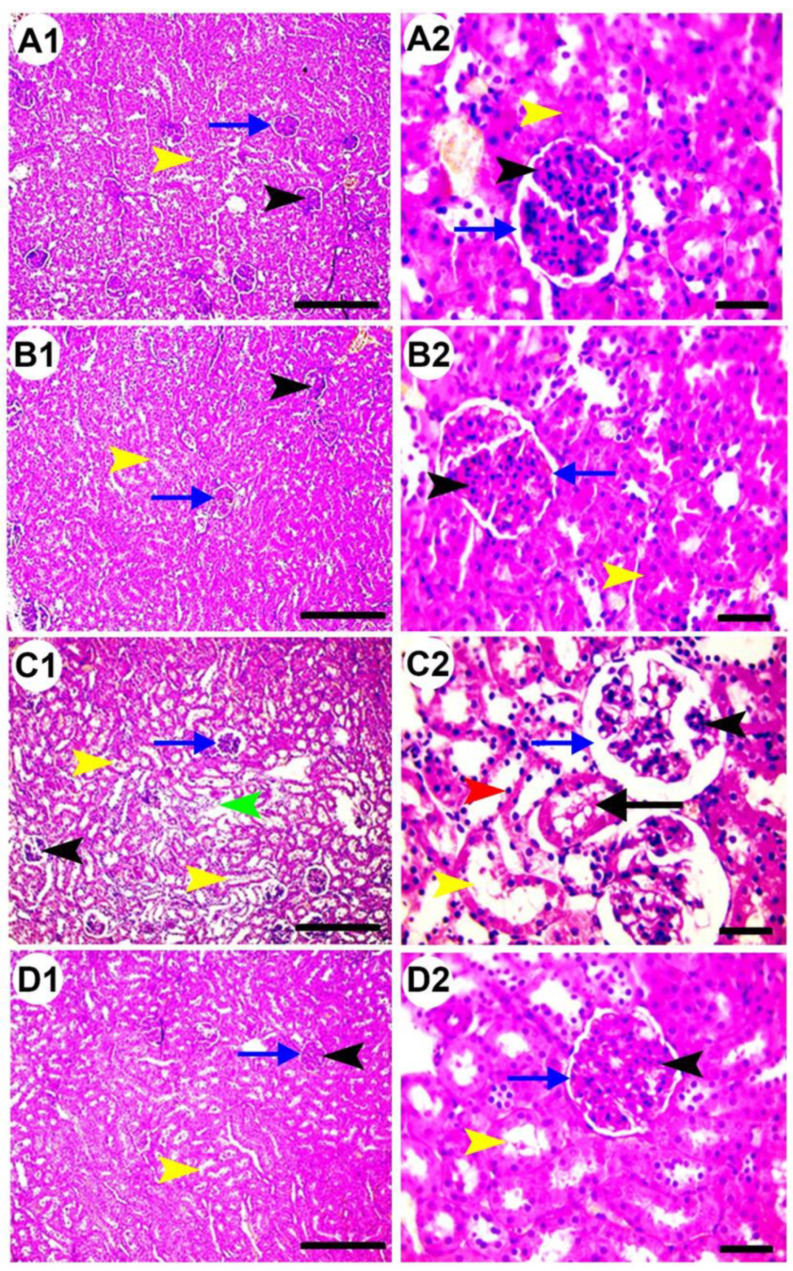

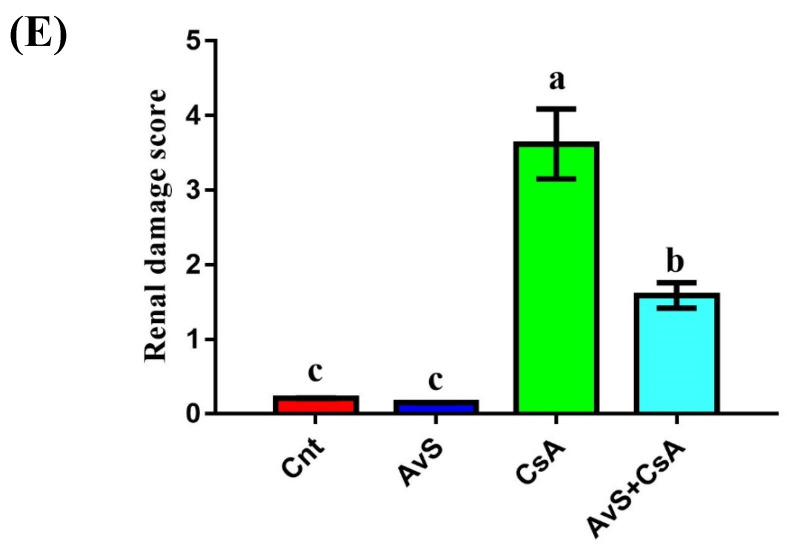
(**A**–**D**) Microscopic pictures of H&E-stained renal sections showing normal glomeruli, tubules, and interstitial tissue from the control (**A1**,**2**) and AvS (**B1**,**2**) groups; tubular dilation, perivascular edema (green arrowhead), hyaline cast (black arrow), pyknotic nuclei (red arrowhead), and widened Bowman’s space from the CsA group (**C1**,**2**); and mild tubular dilation from the AvS + CsA group (**D1**,**2**). Black arrowheads: glomeruli; blue arrows: Bowman’s space; yellow arrowheads: renal tubules. Scale bars: 100 μm for (**A1**,**B1**,**C1**,**D1**); and 50 μm for the remaining images. (**E**) Renal damage score; values are mean ± SEM (*n* = 7/group). Columns with different letters (a–c) are significantly different at *p* < 0.05.

Histological examination of renal tissue sections of control and AvS groups revealed the presence of normal glomeruli (black arrowheads), Bowman’s capsules (blue arrows), and tubules (yellow arrowheads) (Figure 4A,B). In contrast, the administration of CsA resulted in notable histopathological changes in renal corpuscles and tubules (Figure 4C). These changes included shrunken and split glomeruli (black arrowheads), widened Bowman’s space (blue arrows), interstitial and perivascular edema (green arrowhead), tubular dilation (yellow arrowheads), pyknotic nuclei in tubal lining epithelia (red arrowhead), and the accumulation of hyaline cast in the tubal lumen (black arrow). Co-treatment with AvS (AvS + CsA group) reduced the renal damage induced by CsA, resulting in only mild tubular dilation (yellow arrowheads, Figure 4D). The highest renal damage score was given to the CsA group, and the score of the AvS + CsA group was significantly lower (Figure 4E).

## 4. Discussion

For its powerful immunosuppressive properties, CsA is commonly used after organ transplantations. However, its prolonged uses cause many adverse effects, including oxidative-stress-dependent nephrotoxicity [1,2,3,5]. On the other hand, antioxidants are known to protect against oxidative renal damage induced by CsA [34]. The AvS conatins several health-promoting bioactive compounds with antioxidant and anti-inflammatory properties [15,16,17,18,19] that can antagonize the detrimental effects of free radicals and subsequently protect renal cells from damage. Therefore, in this study, we investigated the ameliorative effect of AvS on the nephrotoxicity induced by CsA. We also assessed whether AvS would alter CsA’s immunosuppressive effect. To the best of our knowledge, this is the first study to report that AvS could reduce nephrotoxicity and increase the immunosuppression induced by CsA through, at least in part, modulation of oxidative stress and inflammation and immunity-related markers. We reached this overall conclusion based on the following evidence: co-treatment with AvS resulted in (1) increases in final body weight gain and feed efficiency ratio, (2) reductions in the serum levels of renal function parameters (uric acid, urea, and creatinine), (3) increases in creatinine clearance and renal Na^+^/K^+^ ATPase, (4) a decrease in the renal level of an oxidative stress marker (MDA), (5) increases in the activity of renal antioxidant enzymes (SOD, CAT, GPx), (6) a reduction in DNA damage in renal cells, (7) the downregulation of immunomodulatory and inflammatory genes (*IFN-γ*, *TNFα*, *IL1β*, *MCP1*, *ICAM1*, and *VCAM1*), and (8) improved renal histopathology.

In agreement with Saad [21] and Zizhang et al. [35], treatment with CsA resulted in significant reductions in feed intake (FI), final body weight gain (FBWG), and feed efficiency ratio (FER). Loss of appetite could be attributed to CsA’s ability to penetrate the blood–brain barrier and the subsequent inhibition of the appetite center [36]. CsA and AvS co-treatment improved FI, FBWG, and FER in animals. Interestingly, animals administered AvS alone showed significantly increased FBWG and FER as compared to the control animals. In contrast, Saad [21] found decreases in FBWG and FER following administration of AvS powder to rats. In support to our findings, many other studies have reported a growth-promoting effect for AvS and attributed this effect to the nutritious properties of these seeds, which are rich in vitamins, essential minerals, and amino acids, and several other health-promoting bioactive compounds [15,16,20]. This growth-promoting effect denotes the importance of AvS and its ingredients as a value-added food.

In the present study, treatment with CsA alone resulted in elevated serum levels of renal function biomarkers (uric acid, urea, and creatinine), suggesting severe renal damage. These results are in agreement with many previous reports [37,38]. CsA impairs kidney function by reducing glomerular filtration rate (GFR) and/or inducing tubular damage [34,39]. Creatinine clearance is used as an indicator of plasma renal flow. We found significant decreases in creatinine clearance in urine, Na^+^/K^+^ ATPase activity in kidney, and kidney weight. In accordance with our findings, previous studies also showed similar effects for CsA [40,41]. This functional impairment was followed by structural damage via atrophied glomeruli, widened Bowman’s space, interstitial and perivascular edema, and tubular dilation. Similar histopathological changes were reported by other studies [42,43].

Our results revealed that CsA administration significantly increased the levels of lipid peroxidation biomarker (MDA) and reduced the activities of antioxidant enzymes (SOD, CAT, GPx) in the kidney. Similar results were obtained by Capasso et al. [44]. Oxidative stress commences when the release of reactive oxygen species (ROS) overrides the activities of endogenous antioxidant enzymes, causing damage to various cellular macromolecules, including proteins, lipids, and DNA [31,45,46]. Elevation of renal MDA levels indicates oxidative damage to renal cell membranes, which are rich in lipids [33,47]. Previous studies have shown that CsA can trigger oxidative-stress-dependent nephrotoxicity via induction of endoplasmic reticulum stress and boosting free radical release from mitochondria [8]. The histopathological alterations induced by CsA could be attributed to ROS overproduction, which could cause detrimental effects on the glomerular endothelium and subsequent endothelial dysfunction [48]. Besides, accumulation of hyaline cast within the renal tubules in CsA-treated animals may be due to ROS and lipid peroxidation. The latter can induce cell membrane damage with subsequent shedding of cytoplasmic contents into the lumen that further extend the hyaline cast [49].

We found that co-treatment with AvS powder improves kidney function, structure, and antioxidant status, and inhibits oxidative stress damage in the kidney. Hence, the results suggest that consumption of AvS might have some therapeutic effects for kidney injuries induced by CsA in rats. These results agree with Rao et al. [50] and Abdel Moneim et al. [51], who found that the avocado can act as a nephroprotective agent by inhibiting renal oxidative stress. The AvS’s antioxidant properties could be attributed to its phytochemical ingredients such as phytosterols, triterpenes, fatty acids, furanoic acids, proanthocyanidins, and abscisic acid; and phenolic compounds such as hydrocinnamic acid, catechin, epicatechin, and leucoanthocyanidins [15,16]. Based on GC-MS analysis, we found 21 compounds in the ethanolic extract of the AvS. Most of them were terpenes and esters of fatty acid derivatives (dodecanoic acid, tetradecanoic acid, 9,12-octadecadienoic acid (z, z), 9-octadecenoic acid (z), heptadecanoic acid, 16-methyl,-methyl ester, 9,12-octadecadienoic acid, hexadecadienoic acid, and oleic acid). Richard et al. [52] stated that unsaturated fatty acids can function as antioxidants. Avocado byproducts (seeds and peels) are a good source of phenolic compounds that can inhibit lipid and protein oxidation [53]. Avocado flavonoids could prevent the formation of renal calculi by suppressing the release of ROS, thereby protecting renal cells from oxidative stress damage [54]. Avocado has antioxidant, anti-inflammatory, hypocholesterolemic, and antidiabetic effects [55].

CsA could also induce nephrotoxicity by triggering apoptosis in renal cells [9].  The comet assay results indicate induction of DNA fragmentation in renal cells following treatment with CsA, suggesting a role in apoptosis. Consistently, previous studies also showed that CsA could induce DNA fragmentation, thereby causing chronic organ diseases [56,57]. In contrast, co-treatment with AvS resulted in a significant reduction in DNA damage. The avocado seed extract was shown to have no genotoxic activity [15].

In the present study, we found significant downregulation in the expression levels of immunomodulatory and inflammatory genes (*IFN-γ, TNFa*, *IL1β*, *MCP1*, *ICAM1*, and *VCAM1*) following treatment with CsA. Consistently with those findings, Rincón et al. [58] also reported significant reductions in *IFN-γ* and *TNFa* after treatment with CsA. In contrast, Saad [21] found an increase in TNFα levels in renal tissues after administration of a very high dose of CsA (50 mg/kg). These contradictory results may be attributed to the methods used for quantification of TNFα (Elisa vs. real-time PCR) and the CsA dose (50 mg/kg vs. 5 mg/kg). Therefore, further experiments, including Western blots, will be required to check whether the molecular changes in inflammatory genes are consistent with changes in their proteins. Moreover, the *IL1β* and *MCP1* downregulation by CsA agree with Daull et al. [59,60]. ICAM1 and VCAM1 modulate the interaction between inflammation and immunity [61], and their expression is inhibited by CsA [62]. On the other hand, we found that co-treatment with AvS and CsA maximizes the immunosuppressive effect of CsA, as revealed by lower expression levels of *IFN-γ, TNFa*, *IL1β*, *MCP1*, *ICAM1*, and *VCAM1* in the renal tissues. This implies that AvS has not only antioxidant properties, but also has an anti-inflammatory effect. Indeed, previous studies reported anti-inflammatory effects for avocado and AvS, as revealed by a reduction in cytokine production [13,63]. In contrast, Saad [21] reported an immunostimulant effect for 5% AvS powder following treatment with an overdose of CsA (50 mg/kg).

Avocado seeds, as a waste product, are produced in large amounts during industrial processing, and their unmanageable disposal may cause environmental problems. These seeds are available in bulk at zero cost. Thus, modern approaches are needed to obtain benefits from this waste. For example, these seeds and their ingredients can be used as natural food additives (due to their growth-promoting effect), and in medicine to alleviate CsA’s side effects (due to their antioxidant and anti-inflammatory effects).

The present study lacked results on the effects of different treatments on the concentrations of immunoglobulins in blood, the expression of immunity-related markers for adaptive and innate immunity, and appropriate rejection models of renal transplantation in animals. Moreover, an appropriately designed clinical trial in transplant patients treated with cyclosporin should be carried out to confirm our results in a relevant human model before translation to the clinic.

## 5. Conclusions

Many natural products relieved CsA’s oxidative stress effects, but it is still unknown whether these natural products could modulate CsA’s immunosuppressive effect. To the best of our knowledge, this is the first study to report that AvS treatment not only reduces the oxidative-stress-dependent nephrotoxicity induced by CsA, but also modulates the expression of inflammation and immunity-related genes, which could improve CsA’s immunosuppressive effect. This was accomplished by decreasing oxidative stress, apoptosis, and inflammation, all of which were manifested in the enhanced biochemical parameters and diminished renal damage scores. AvS could therefore potentially be a therapeutic choice for patients with CsA-induced nephrotoxicity. However, further experiments are required to unveil the exact molecular pathways by which AvS exerts this beneficial effect, and to determine whether AvS is clinically applicable.

## Figures and Tables

**Table 1:** Formulations of the diets—basal or supplemented with 5% avocado seed powder.

Ingredients (g/Kg)	Basal Diet	Basal Diet with 5% Avocado Seed Powder
Casein (>85% protein)	120.0	120.0
Corn oil	70.0	70.0
Mineral mix	35.0	35.0
Vitamin mix	10.0	10.0
L- cystine	3.0	3.0
Choline bitartrate	2.5	2.5
Wheat bran	50.0	50.0
Sucrose	100.0	100.0
Corn starch	609.5	559.5
Avocado seeds powder	--	50.0

**Table 2:** Primers used for real-time PCR.

Gene	Forward Primer	Reverse Primer	Accession Number
*TNFα*	GCATGATCCGCGACGTGGAA	AGATCCATGCCGTTGGCCAG	NM_012675.3
*IL1β*	CACCTCTCAAGCAGAGCACAG	GGGTTCCATGGTGAAGTCAAC	NM_031512.2
*MCP1*	TCGCTTCTGACACCATGCA	TGCTACAGGCAGCAAATGTGA	M57441.1
*IFN-γ*	GATCCAGCACAAAGCTGTCA	GACTCCTTTTCCGCTTCCTT	AF010466.1
*ICAM1*	AGATCACATTCACGGTGCTG	CTTCAGAGGCAGGAAACAGG	NM_012967.1
*VCAM1*	TGCACGGTCCCTAATGTGTA	TGCCAATTTCCTCCCTTAAA	NM_012889.2
*GAPDH*	CAACTCCCTCAAGATTGTCAGCAA	GGCATGGACTGTGGTCATGA	NM_017008.4

**Table 3:** Chemical compounds, retention time (RT), and constituents of the avocado seed as detected by GC-MS.

RT	Compound Name	Area %	Molecular Formula	Molecular Weight
14.23	7-Hexadecene	1.41	C16H32	224
14.60	10-Undecenal	2.32	C11H20O	168
17.25	13-Oxabicyclo[1 0.1.0]tridecan	4.58	C12H22O	182
19.94	Tridecanal	2.22	C13H26O	198
20.28	Dodecanoic acid, methyl ester	5.53	C13H26O2	214
21.64	5Oxatricyclo[8.2.0.0(4,6)]dodecane, 12-trimethyl-9-methylene	1.32	C15H24O	220
22.27	cis-9-Hexadecenal	1.71	C16H30O	238
22.37	Tetradecanal	4.34	C14H28O	212
24.20	Cyclopentadecanone	3.33	C15H28O	224
24.90	Tetradecanoic acid, methyl ester	3.37	C15H30O2	242
26.42	Z,Z-4,15-Octadecadien-1-ol acetate	26.06	C20H36O2	308
27.12	Aromadendrene oxide-(1)	8.63	C15H24O	220
29.11	Hexadecanoic acid, methyl ester	7.28	C17H34O2	270
30.67	Oleic Acid	1.31	C18H34O2	282
31.88	9-Octadecenoic acid (z)	3.47	C18H34O2	282
32.33	9,12-Octadecadienoic acid (z,z)-, methyl ester	1.13	C19H34O2	294
32.45	9-Octadecenoic acid (Z)-, methyl ester	3.77	C19H36O2	296
32.94	Heptadecanoic acid, 16-methyl-, methyl ester	1.26	C19H38O2	298
33.67	9,12-Octadecadienoic acid	9.32	C18H32O2	280
34.92	7-Methyl-Z-tetradecen-1-ol acetate	5.71	C17H32O2	268
35.53	Hexadecadienoic acid, methyl ester	1.94	C17H30O2	266

**Table 4:** Effects of avocado seed powder on growth-related parameters.

Groups	FBWG (g)	Total FI (g)	FER
Cnt	60.00 ± 3.28 ^b^	501.00 ± 12.79 ^a^	0.120 ± 0.006 ^b^
AvS	73.33 ± 3.19 ^a^	475.00 ± 10.43 ^a^	0.154 ± 0.002 ^a^
CsA	30.33 ± 2.03 ^d^	351.67 ± 8.67 ^c^	0.086 ± 0.007 ^d^
AvS + CsA	47.00 ± 2.61 ^c^	437.00 ± 11.89 ^b^	0.108 ± 0.004 ^c^

Means with different superscript letters (a–d) in the same column are significantly different at *p* < 0.05. Data are presented as mean ± SEM (*n* = 7 animals in each group). Cnt: control group; AvS: avocado seed powder-treated group; CsA: cyclosporine A-treated group; AvS + CsA: cyclosporine A and avocado seed powder-cotreated group. FBWG, final body weight gain; FI, feed intake; FER, feed efficiency ratio.

**Table 5:** Effects of avocado seed powder on the serum levels of kidney function biomarkers in rats.

Groups	Uric Acid (mg/dL)	Urea (mg/dL)	Creatinine (mg/dL)
Cnt	2.61 ± 0.10 ^c^	17.63 ± 0.76 ^c^	0.72 ± 0.03 ^c^
AvS	2.13 ± 0.12 ^d^	14.31 ± 0.63 ^d^	0.64 ± 0.02 ^d^
CsA	6.56 ± 0.21 ^a^	74.23 ± 2.18 ^a^	1.46 ± 0.06 ^a^
AvS + CsA	3.62 ± 0.11 ^b^	32.35 ± 1.06 ^b^	0.93 ± 0.04 ^b^

Means with different superscript letters (a–d) in the same column are significantly different at *p* < 0.05. Data are presented as mean ± SEM (*n* = 7/group).

**Table 6:** Effects of avocado seed powder on oxidant/antioxidant status of kidney.

Groups	MDA (nmol/g Tissue)	SOD (U/g Tissue)	CAT (U/g Tissue)	GPx (U/g Tissue)
Cnt	11.37 ± 0.73 ^c^	3.75 ± 0.24 ^a^	3.04 ± 0.19 ^a^	115.75 ± 7.51 ^b^
AvS	9.70 ± 0.54 ^d^	3.94 ± 0.28 ^a^	3.15 ± 0.17 ^a^	138.11 ± 7.82 ^a^
CsA	35.68 ± 2.72 ^a^	1.21 ± 0.09 ^c^	1.46 ± 0.08 ^c^	37.30 ± 2.36 ^d^
AvS + CsA	18.24 ± 0.91 ^b^	2.45 ± 0.14 ^b^	2.25 ± 0.09 ^b^	92.73 ± 6.00 ^c^

Means with different superscript letters (a–d) in the same column are significantly different at *p* < 0.05. Data are presented as mean ± SEM (*n* = 7/group).

**Table 7:** Effects of avocado seed powder on creatinine clearance, Na^+^/K^+^ ATPase, and kidney weight.

Groups	Creatinine Clearance (mL/min/100 g Body wt)	Na^+^/K^+^ ATPase (ng/mg)	kidney Weight (g)
Cnt	1.6 ± 0.04 ^a^	0.85 ± 0.02 ^a^	1.66 ± 0.05 ^bc^
AvS	1.14 ± 0.02 ^c^	0.79 ± 0.005 ^b^	1.93 ± 0.02 ^a^
CsA	0.82 ± 0.03 ^d^	0.14 ± 0.004 ^d^	1.26 ± 0.05 ^d^
AvS + CsA	1.25 ± 0.04 ^b^	0.55 ± 0.03 ^c^	1.60 ± 0.03 ^c^

Means with different superscript letters (a–d) in the same column are significantly different at *p* < 0.05. Data are presented as mean ± SEM (*n* = 7/group).

**Table 8:** Effects of AvS powder on DNA damage, as detected by comet assay in rat kidneys.

Group	Tailed %	Untailed %	Tail Length (µm)	Tail DNA%	Tail Moment *
Cnt	2	98	1.65 ± 0.11 ^c^	1.10	1.82
AvS	4	96	1.89 ± 0.12 ^c^	1.75	3.31
CsA	18	82	5.63 ± 0.28 ^a^	4.50	25.34
AvS + CsA	11	89	3.95 ± 0.19 ^b^	3.24	12.80

Data are expressed as mean ± SEM (*n* = 7/group). Different superscript letters (a–c) in the same column of tail length show significance differences at *p* < 0.05. * Tail moment is the tail DNA (%) × tail length (µm).

## Data Availability

The data supporting the present findings are contained within the manuscript.
